# (3*S*,3a*S*,6*R*,6a*R*)-2-Oxohexa­hydro­furo[3,2-*b*]furan-3,6-diyl dibenzoate

**DOI:** 10.1107/S160053681302391X

**Published:** 2013-09-04

**Authors:** Vincenzo Piccialli, Giorgia Oliviero, Sabrina Zaccaria, Roberto Centore, Angela Tuzi

**Affiliations:** a Dipartimento di Scienze Chimiche, Università degli Studi di Napoli ’Federico II’, Complesso di Monte S. Angelo, Via Cinthia, 80126 Napoli, Italy; b Dipartimento di Farmacia, Università degli Studi di Napoli ’Federico II’, Via D. Montesano 49, 80131 Napoli, Italy

## Abstract

The title compound, C_20_H_16_O_7_, contains a *cis*-fused γ-lactone tetra­hydro­furan ring system functionalized with two benzo­yloxy groups. Both rings adopt an envelope conformation. The mol­ecule assumes an elongated shape and exibits non-crystallographic *C*_2_ symmetry. The benzo­yloxy groups are almost planar [maximum deviations of 0.0491 (15) and 0.0336 (17) Å for the O atoms] and their mean planes are inclined to one another by 16.51 (4)°. The crystal packing features weak C—H⋯O inter­actions. The aryl groups of adjacent mol­ecules are parallel shifted with face-to-face contacts and a shortest inter­molecular C⋯C distance of 3.482 (4) Å.

## Related literature

For the use of carbohydrate in the synthesis of complex natural chiral substances, see: Hanessian (1993 ▶). For mannitol as chiral reagent and as a precursor of biologically active derivatives, see: Masaki *et al.* (1999 ▶); Lohray *et al.* (1999 ▶). For oxidative processes mediated by transition metal oxo-species, see: De Champdorè *et al.*. (1998 ▶); Piccialli (2007 ▶); Piccialli, Oliviero *et al.* (2013 ▶); Piccialli, Tuzi *et al.* (2013 ▶). For the catalytic use of chloro­chromato­periodate, see: Piccialli, D’Errico *et al.* (2013 ▶); Piccialli *et al.* (2012 ▶). For the synthesis of the precursor, see: Hockett *et al.* (1946 ▶).
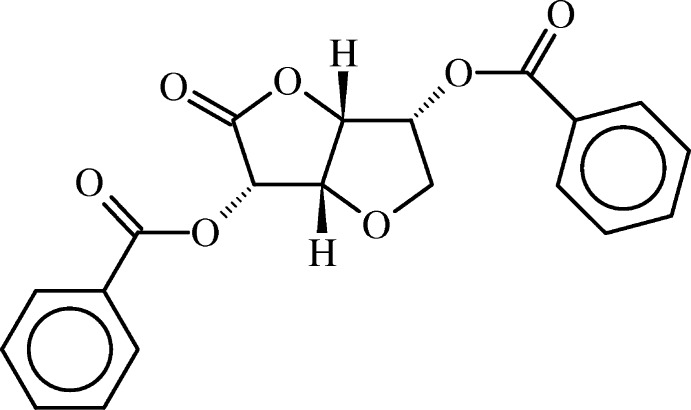


## Experimental

### 

#### Crystal data

C_20_H_16_O_7_
*M* *_r_* = 368.33
Orthorhombic, 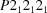

*a* = 7.4870 (7) Å
*b* = 10.2050 (14) Å
*c* = 22.232 (2) Å
*V* = 1698.6 (3) Å^3^
*Z* = 4
Mo *K*α radiation
μ = 0.11 mm^−1^
*T* = 173 K
0.50 × 0.40 × 0.08 mm

#### Data collection

Bruker–Nonius KappaCCD diffractometer
Absorption correction: multi-scan (*SADABS*; Bruker, 2001 ▶) *T*_min_ = 0.947, *T*_max_ = 0.991
8078 measured reflections
2108 independent reflections
1797 reflections with *I* > 2σ(*I*)
*R*_int_ = 0.032

#### Refinement

*R*[*F* ^2^ > 2σ(*F* ^2^)] = 0.034
*wR*(*F* ^2^) = 0.078
*S* = 1.07
2108 reflections
244 parameters
H-atom parameters constrained
Δρ_max_ = 0.16 e Å^−3^
Δρ_min_ = −0.19 e Å^−3^

### 

Data collection: *COLLECT* (Nonius, 1999 ▶); cell refinement: *DIRAX/LSQ* (Duisenberg *et al.*, 2000 ▶); data reduction: *EVALCCD* (Duisenberg *et al.*, 2003 ▶); program(s) used to solve structure: *SIR97* (Altomare *et al.*, 1999 ▶); program(s) used to refine structure: *SHELXL97* (Sheldrick, 2008 ▶); molecular graphics: *ORTEP-3 for Windows* (Farrugia, 2012 ▶) and *Mercury* (Macrae *et al.*, 2006 ▶); software used to prepare material for publication: *WinGX* (Farrugia, 2012 ▶).

## Supplementary Material

Crystal structure: contains datablock(s) global, I. DOI: 10.1107/S160053681302391X/zp2007sup1.cif

Structure factors: contains datablock(s) I. DOI: 10.1107/S160053681302391X/zp2007Isup2.hkl

Supplementary material file. DOI: 10.1107/S160053681302391X/zp2007Isup3.cml

Additional supplementary materials:  crystallographic information; 3D view; checkCIF report

## Figures and Tables

**Table 1:** Hydrogen-bond geometry (Å, °)

*D*—H⋯*A*	*D*—H	H⋯*A*	*D*⋯*A*	*D*—H⋯*A*
C2—H2⋯O6^i^	1.00	2.34	2.974 (2)	121
C3—H3⋯O4^ii^	1.00	2.51	3.356 (3)	142
C10—H10⋯O7^iii^	0.95	2.48	3.353 (3)	154

Symmetry codes: (i) 
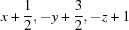
; (ii) 
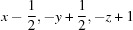
; (iii) 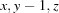.

**Fig. 1.**
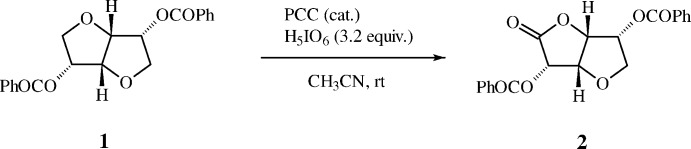
Synthesis of the title compound by chlorochromatoperiodate (CCP) catalyzed oxidation of 1.

**Fig. 2.**
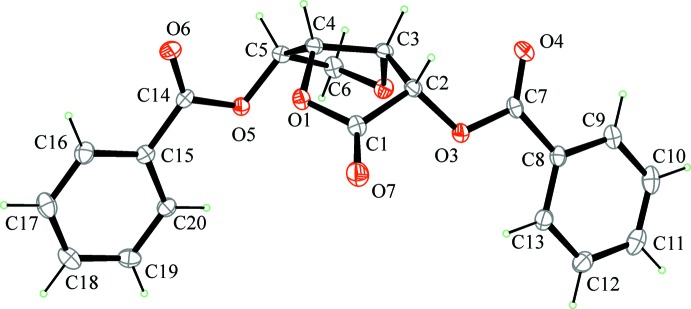
ORTEP view of the the title compound. Thermal ellipsoids are drawn at 30% probability level.

**Fig. 3.**
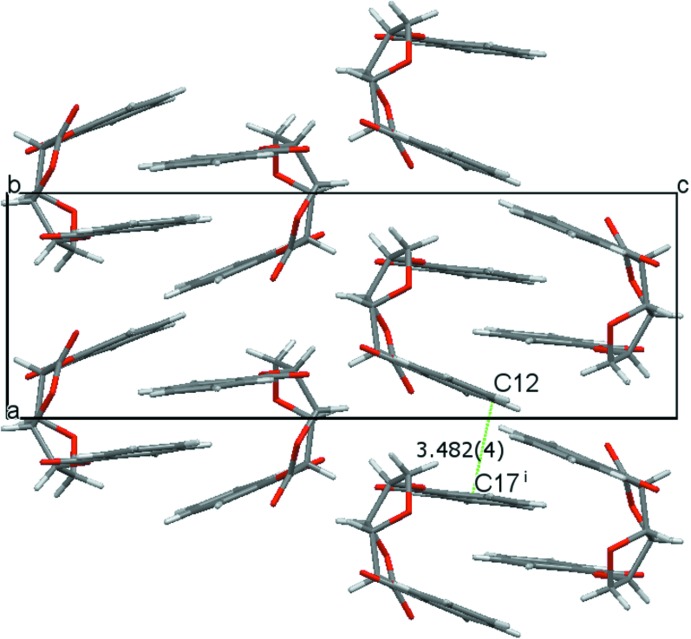
Crystal packing viewed along b axis. Shortest aryl intermolecular C···C distance is shown as dashed line (i = 1 + x, -1 + y, z).

## supplementary crystallographic information

### 1. Comment

D-mannitol plays an important role in organic synthesis as a readily available chiral building block (Hanessian, 1993). In addition, mannitol and its derivatives are widely used as chiral reagents and chiral auxiliaries (Masaki *et al.*, 1999) and can be transformed into biologically active and pharmaceutically important compounds (Lohray *et al.*, 1999). As a part of our ongoing interest in oxidative processes mediated by transition metals oxo-species (De Champdorè *et al.*. 1998; Piccialli, Oliviero, Borbone *et al.*, 2013; Piccialli, Tuzi, Oliviero *et al.*, 2013; Piccialli, 2007), and in particular in the synthesis of new THF-containing compounds, we recently focused on the catalytic use of chlorochromatoperiodate (*CCP*), a powerful oxidizing reagent generated by the condensation of pyridinium chlorochromate (PCC) and periodic acid (Piccialli, D'Errico, Borbone *et al.*, 2013; Piccialli, Zaccaria, Oliviero *et al.*, 2012).

The title compound, C_20_H_18_O_7_, contains a functionalized *cis*–fused γ-lactone-tetrahydrofuran ring system, substituted at C2 and C5 positions with benzoyloxy groups. Both rings adopt an envelope conformation with O2 and C3 at the flap. The molecule assumes an elongated shape and exibits a C_2_ symmetry of the benzoyloxy groups (Fig.2). The benzoyloxy groups are almost planar (maximum deviation from least square plane (O3/O4/C7—C13) is -0.0491 (15) Å for O3; maximum deviation from least square plane (O5/O6/C14—C20) is 0.0336 (17) Å for O6) and their mean planes are inclined with respect to each other by 16.51 (4)°. No strong H–bonding donor groups are present in the molecule. The crystal packing (Fig.3) is stabilized by weak intermolecular CH···O interactions. Aryl groups of adjacent molecules are parallel shifted with face-to-face contacts (shortest intermolecular distance is C12···C17^i^ = 3.482 (4) Å, i = 1 + *x*, -1 + *y*, *z*).

### 2. Experimental

The title compound 2 has been synthesized by oxidation of bis-tetrahydrofuran 1 with chlorochromatoperiodate (*CCP*) according to the scheme in Fig. 1. Compound 1 has been obtained from mannitol according to literature (Hockett *et al.*, 1946). Since the oxidative process does not affect any of the chiral centres of the molecule, the absolute configuration of 2 matches that of mannitol. Crystals suitable for X-ray analysis were obtained by recrystallization of 2 from methanol.

Compound 2: ^1^H-NMR (200 MHz, CDCl_3_) 8.23–8.03 (4*H*, m), 7.62 (2*H*, t, *J*= 7.6 Hz), 7.48 (4*H*, t, *J*= 7.5 Hz), 5.63 (1*H*, d, *J*= 5.6 Hz, H-2), 5.58–5.34 (2*H*, overlapped multiplets, H-4 and H-5), 5.06 (1*H*, dd, *J*= 5.6, 4.0 Hz, H-3), 4.33 (1*H*, dd, *J*= 9.3, 6.9 Hz, Ha-6), 4.03 (1*H*, dd, *J*= 9.3, 7.8 Hz, Hb-6); ^13^C-NMR (50 MHz, CDCl_3_) 170.4, 165.8, 165.3, 133.9, 133.7, 130.2, 130.0, 129.8, 128.65, 128.56, 128.4, 128.2, 78.6, 76.1, 72.9, 69.4, 69.2.

### 3. Refinement

All H atoms were generated stereochemically and refined by the riding model with *U*_iso_=1.2×*U*_eq_ of the carrier atom. In the absence of strong anomalous scatterer the Flack parameter is not meaningful. Data were merged using MERG 3 instruction and the absolute configuration was assigned on the basis of the configuration of its percursor.

### Crystal data

**Table:**

C_20_H_16_O_7_	*F*(000) = 768
*M**_r_* = 368.33	*D*_x_ = 1.440 Mg m^−^^3^
Orthorhombic, *P*2_1_2_1_2_1_	Mo *K*α radiation, λ = 0.71073 Å
Hall symbol: P 2ac 2ab	Cell parameters from 119 reflections
*a* = 7.4870 (7) Å	θ = 3.8–22.3°
*b* = 10.2050 (14) Å	µ = 0.11 mm^−^^1^
*c* = 22.232 (2) Å	*T* = 173 K
*V* = 1698.6 (3) Å^3^	Block, white
*Z* = 4	0.50 × 0.40 × 0.08 mm

### Data collection

**Table:**

Bruker–Nonius KappaCCD diffractometer	2108 independent reflections
Radiation source: normal-focus sealed tube	1797 reflections with *I* > 2σ(*I*)
Graphite monochromator	*R*_int_ = 0.032
Detector resolution: 9 pixels mm^-1^	θ_max_ = 27.5°, θ_min_ = 3.3°
CCD rotation images, thick slices scans	*h* = −9→5
Absorption correction: multi-scan (*SADABS*; Bruker, 2001)	*k* = −12→13
*T*_min_ = 0.947, *T*_max_ = 0.991	*l* = −28→28
8078 measured reflections	

### Refinement

**Table:**

Refinement on *F*^2^	Primary atom site location: structure-invariant direct methods
Least-squares matrix: full	Secondary atom site location: difference Fourier map
*R*[*F*^2^ > 2σ(*F*^2^)] = 0.034	Hydrogen site location: inferred from neighbouring sites
*wR*(*F*^2^) = 0.078	H-atom parameters constrained
*S* = 1.07	*w* = 1/[σ^2^(*F*_o_^2^) + (0.0383*P*)^2^ + 0.2376*P*] where *P* = (*F*_o_^2^ + 2*F*_c_^2^)/3
2108 reflections	(Δ/σ)_max_ < 0.001
244 parameters	Δρ_max_ = 0.16 e Å^−^^3^
0 restraints	Δρ_min_ = −0.19 e Å^−^^3^

### Special details

**Table:**

Geometry. All e.s.d.'s (except the e.s.d. in the dihedral angle between two l.s. planes) are estimated using the full covariance matrix. The cell e.s.d.'s are taken into account individually in the estimation of e.s.d.'s in distances, angles and torsion angles; correlations between e.s.d.'s in cell parameters are only used when they are defined by crystal symmetry. An approximate (isotropic) treatment of cell e.s.d.'s is used for estimating e.s.d.'s involving l.s. planes.
Refinement. Refinement of *F*^2^ against ALL reflections. The weighted *R*-factor *wR* and goodness of fit *S* are based on *F*^2^, conventional *R*-factors *R* are based on *F*, with *F* set to zero for negative *F*^2^. The threshold expression of *F*^2^ > σ(*F*^2^) is used only for calculating *R*-factors(gt) *etc*. and is not relevant to the choice of reflections for refinement. *R*-factors based on *F*^2^ are statistically about twice as large as those based on *F*, and *R*- factors based on ALL data will be even larger.

### Fractional atomic coordinates and isotropic or equivalent isotropic displacement parameters (Å^2^)

**Table:**

	*x*	*y*	*z*	*U*_iso_*/*U*_eq_	
C1	0.7473 (3)	0.5893 (2)	0.57766 (9)	0.0274 (5)	
C2	0.7006 (3)	0.45523 (19)	0.55289 (9)	0.0256 (5)	
H2	0.7605	0.4416	0.5132	0.031*	
C3	0.5016 (3)	0.46156 (19)	0.54459 (9)	0.0248 (5)	
H3	0.4587	0.4085	0.5096	0.030*	
C4	0.4649 (3)	0.60851 (19)	0.53746 (9)	0.0260 (5)	
H4	0.4546	0.6345	0.4942	0.031*	
C5	0.2879 (3)	0.6297 (2)	0.57190 (9)	0.0276 (5)	
H5	0.1904	0.6574	0.5439	0.033*	
C6	0.2490 (3)	0.4957 (2)	0.59859 (11)	0.0324 (5)	
H6A	0.2004	0.5045	0.6398	0.039*	
H6B	0.1612	0.4480	0.5735	0.039*	
C7	0.7436 (3)	0.2341 (2)	0.57841 (9)	0.0277 (5)	
C8	0.8031 (3)	0.1437 (2)	0.62616 (9)	0.0267 (5)	
C9	0.7809 (3)	0.0097 (2)	0.61663 (11)	0.0350 (6)	
H9	0.7348	−0.0215	0.5795	0.042*	
C10	0.8264 (4)	−0.0772 (2)	0.66150 (12)	0.0425 (7)	
H10	0.8131	−0.1687	0.6551	0.051*	
C11	0.8912 (4)	−0.0320 (2)	0.71559 (12)	0.0425 (6)	
H11	0.9204	−0.0925	0.7465	0.051*	
C12	0.9142 (4)	0.1007 (2)	0.72531 (11)	0.0369 (6)	
H12	0.9591	0.1314	0.7627	0.044*	
C13	0.8714 (3)	0.1880 (2)	0.68034 (10)	0.0320 (5)	
H13	0.8889	0.2793	0.6865	0.038*	
C14	0.3130 (3)	0.8490 (2)	0.60421 (9)	0.0271 (5)	
C15	0.3326 (3)	0.9379 (2)	0.65625 (9)	0.0256 (5)	
C16	0.3399 (3)	1.0720 (2)	0.64556 (10)	0.0330 (5)	
H16	0.3290	1.1049	0.6058	0.040*	
C17	0.3629 (4)	1.1568 (2)	0.69311 (11)	0.0381 (6)	
H17	0.3679	1.2485	0.6860	0.046*	
C18	0.3788 (4)	1.1098 (2)	0.75109 (10)	0.0382 (6)	
H18	0.3943	1.1689	0.7837	0.046*	
C19	0.3722 (4)	0.9768 (2)	0.76157 (10)	0.0349 (6)	
H19	0.3843	0.9443	0.8014	0.042*	
C20	0.3479 (3)	0.8907 (2)	0.71458 (9)	0.0282 (5)	
H20	0.3418	0.7992	0.7221	0.034*	
O1	0.6118 (2)	0.67337 (13)	0.56690 (6)	0.0276 (4)	
O2	0.4158 (2)	0.42772 (14)	0.59961 (6)	0.0294 (4)	
O3	0.7629 (2)	0.36076 (13)	0.59524 (6)	0.0272 (4)	
O4	0.6838 (3)	0.20348 (14)	0.53016 (7)	0.0386 (4)	
O5	0.3071 (2)	0.72131 (13)	0.62053 (6)	0.0268 (4)	
O6	0.3053 (3)	0.88274 (15)	0.55255 (6)	0.0438 (5)	
O7	0.8798 (2)	0.62237 (15)	0.60320 (8)	0.0403 (4)	

### Atomic displacement parameters (Å^2^)

**Table:**

	*U*^11^	*U*^22^	*U*^33^	*U*^12^	*U*^13^	*U*^23^
C1	0.0279 (12)	0.0252 (11)	0.0292 (11)	−0.0009 (10)	0.0049 (10)	−0.0007 (8)
C2	0.0339 (14)	0.0222 (10)	0.0206 (9)	0.0002 (10)	0.0014 (9)	−0.0017 (8)
C3	0.0318 (13)	0.0251 (11)	0.0177 (9)	−0.0011 (10)	−0.0012 (9)	−0.0012 (8)
C4	0.0293 (13)	0.0264 (11)	0.0221 (10)	−0.0009 (10)	−0.0022 (9)	−0.0007 (8)
C5	0.0300 (13)	0.0289 (11)	0.0239 (10)	0.0003 (10)	−0.0025 (10)	−0.0022 (9)
C6	0.0274 (13)	0.0300 (12)	0.0397 (12)	−0.0024 (10)	0.0035 (11)	−0.0020 (9)
C7	0.0276 (12)	0.0250 (11)	0.0304 (11)	0.0039 (10)	0.0073 (10)	−0.0055 (8)
C8	0.0243 (12)	0.0254 (11)	0.0304 (10)	0.0043 (9)	0.0051 (10)	−0.0006 (9)
C9	0.0356 (15)	0.0260 (12)	0.0434 (13)	0.0029 (11)	0.0049 (12)	−0.0042 (9)
C10	0.0460 (17)	0.0242 (12)	0.0572 (16)	0.0052 (12)	0.0121 (14)	0.0036 (11)
C11	0.0402 (15)	0.0370 (13)	0.0504 (14)	0.0098 (12)	0.0072 (14)	0.0157 (12)
C12	0.0339 (15)	0.0401 (13)	0.0368 (12)	0.0060 (12)	−0.0029 (11)	0.0037 (10)
C13	0.0300 (13)	0.0286 (12)	0.0373 (12)	0.0045 (10)	−0.0009 (11)	0.0006 (9)
C14	0.0267 (13)	0.0269 (11)	0.0278 (10)	0.0039 (10)	−0.0028 (10)	0.0047 (9)
C15	0.0216 (12)	0.0292 (11)	0.0260 (10)	0.0011 (9)	0.0022 (9)	0.0012 (8)
C16	0.0365 (15)	0.0301 (11)	0.0323 (11)	−0.0007 (11)	0.0047 (10)	0.0066 (9)
C17	0.0419 (16)	0.0269 (11)	0.0455 (13)	−0.0077 (11)	0.0102 (12)	−0.0020 (10)
C18	0.0389 (15)	0.0370 (13)	0.0388 (12)	−0.0091 (12)	0.0047 (12)	−0.0110 (10)
C19	0.0364 (15)	0.0432 (13)	0.0252 (10)	−0.0033 (12)	0.0007 (10)	−0.0004 (10)
C20	0.0284 (13)	0.0292 (11)	0.0270 (10)	−0.0002 (10)	0.0008 (9)	0.0040 (9)
O1	0.0274 (9)	0.0222 (7)	0.0332 (8)	−0.0002 (7)	0.0008 (7)	−0.0023 (6)
O2	0.0310 (9)	0.0285 (7)	0.0289 (7)	−0.0023 (7)	0.0029 (7)	0.0048 (6)
O3	0.0341 (9)	0.0211 (7)	0.0263 (7)	0.0024 (6)	−0.0015 (7)	−0.0005 (6)
O4	0.0540 (12)	0.0322 (8)	0.0297 (8)	0.0067 (8)	−0.0043 (8)	−0.0092 (6)
O5	0.0332 (9)	0.0238 (7)	0.0233 (7)	0.0034 (7)	−0.0001 (7)	−0.0001 (6)
O6	0.0734 (14)	0.0335 (8)	0.0245 (7)	0.0041 (9)	−0.0101 (9)	0.0054 (6)
O7	0.0302 (10)	0.0341 (9)	0.0568 (10)	−0.0025 (8)	−0.0076 (9)	−0.0079 (8)

### Geometric parameters (Å, º)

**Table:**

C1—O7	1.191 (3)	C9—C10	1.377 (3)
C1—O1	1.350 (3)	C9—H9	0.9500
C1—C2	1.516 (3)	C10—C11	1.376 (4)
C2—O3	1.426 (2)	C10—H10	0.9500
C2—C3	1.502 (3)	C11—C12	1.381 (4)
C2—H2	1.0000	C11—H11	0.9500
C3—O2	1.424 (2)	C12—C13	1.377 (3)
C3—C4	1.533 (3)	C12—H12	0.9500
C3—H3	1.0000	C13—H13	0.9500
C4—O1	1.441 (3)	C14—O6	1.200 (2)
C4—C5	1.545 (3)	C14—O5	1.353 (2)
C4—H4	1.0000	C14—C15	1.477 (3)
C5—O5	1.436 (2)	C15—C20	1.388 (3)
C5—C6	1.519 (3)	C15—C16	1.390 (3)
C5—H5	1.0000	C16—C17	1.377 (3)
C6—O2	1.428 (3)	C16—H16	0.9500
C6—H6A	0.9900	C17—C18	1.381 (3)
C6—H6B	0.9900	C17—H17	0.9500
C7—O4	1.204 (3)	C18—C19	1.378 (3)
C7—O3	1.354 (2)	C18—H18	0.9500
C7—C8	1.475 (3)	C19—C20	1.377 (3)
C8—C13	1.385 (3)	C19—H19	0.9500
C8—C9	1.394 (3)	C20—H20	0.9500
			
O7—C1—O1	121.98 (19)	C10—C9—H9	120.3
O7—C1—C2	128.4 (2)	C8—C9—H9	120.3
O1—C1—C2	109.61 (19)	C11—C10—C9	120.3 (2)
O3—C2—C3	115.75 (18)	C11—C10—H10	119.9
O3—C2—C1	107.13 (16)	C9—C10—H10	119.9
C3—C2—C1	103.58 (18)	C10—C11—C12	120.6 (2)
O3—C2—H2	110.0	C10—C11—H11	119.7
C3—C2—H2	110.0	C12—C11—H11	119.7
C1—C2—H2	110.0	C13—C12—C11	119.5 (2)
O2—C3—C2	109.37 (17)	C13—C12—H12	120.3
O2—C3—C4	104.18 (16)	C11—C12—H12	120.3
C2—C3—C4	103.46 (18)	C12—C13—C8	120.4 (2)
O2—C3—H3	113.0	C12—C13—H13	119.8
C2—C3—H3	113.0	C8—C13—H13	119.8
C4—C3—H3	113.0	O6—C14—O5	122.07 (19)
O1—C4—C3	105.38 (17)	O6—C14—C15	125.32 (19)
O1—C4—C5	111.41 (15)	O5—C14—C15	112.60 (16)
C3—C4—C5	103.87 (17)	C20—C15—C16	119.9 (2)
O1—C4—H4	111.9	C20—C15—C14	121.81 (18)
C3—C4—H4	111.9	C16—C15—C14	118.32 (19)
C5—C4—H4	111.9	C17—C16—C15	119.5 (2)
O5—C5—C6	108.12 (16)	C17—C16—H16	120.2
O5—C5—C4	112.23 (18)	C15—C16—H16	120.2
C6—C5—C4	103.39 (18)	C16—C17—C18	120.6 (2)
O5—C5—H5	110.9	C16—C17—H17	119.7
C6—C5—H5	110.9	C18—C17—H17	119.7
C4—C5—H5	110.9	C19—C18—C17	119.8 (2)
O2—C6—C5	106.02 (19)	C19—C18—H18	120.1
O2—C6—H6A	110.5	C17—C18—H18	120.1
C5—C6—H6A	110.5	C20—C19—C18	120.3 (2)
O2—C6—H6B	110.5	C20—C19—H19	119.8
C5—C6—H6B	110.5	C18—C19—H19	119.8
H6A—C6—H6B	108.7	C19—C20—C15	119.9 (2)
O4—C7—O3	122.27 (19)	C19—C20—H20	120.0
O4—C7—C8	126.27 (19)	C15—C20—H20	120.0
O3—C7—C8	111.46 (17)	C1—O1—C4	111.24 (15)
C13—C8—C9	119.8 (2)	C3—O2—C6	105.25 (16)
C13—C8—C7	122.24 (19)	C7—O3—C2	115.35 (16)
C9—C8—C7	117.9 (2)	C14—O5—C5	115.34 (15)
C10—C9—C8	119.5 (2)		
			
O7—C1—C2—O3	39.4 (3)	C7—C8—C13—C12	175.6 (2)
O1—C1—C2—O3	−140.40 (18)	O6—C14—C15—C20	−177.3 (3)
O7—C1—C2—C3	162.3 (2)	O5—C14—C15—C20	1.7 (3)
O1—C1—C2—C3	−17.6 (2)	O6—C14—C15—C16	1.2 (4)
O3—C2—C3—O2	31.1 (2)	O5—C14—C15—C16	−179.9 (2)
C1—C2—C3—O2	−85.81 (19)	C20—C15—C16—C17	0.2 (4)
O3—C2—C3—C4	141.68 (16)	C14—C15—C16—C17	−178.3 (2)
C1—C2—C3—C4	24.7 (2)	C15—C16—C17—C18	0.0 (4)
O2—C3—C4—O1	90.05 (19)	C16—C17—C18—C19	0.2 (4)
C2—C3—C4—O1	−24.3 (2)	C17—C18—C19—C20	−0.6 (4)
O2—C3—C4—C5	−27.2 (2)	C18—C19—C20—C15	0.9 (4)
C2—C3—C4—C5	−141.55 (16)	C16—C15—C20—C19	−0.6 (4)
O1—C4—C5—O5	7.1 (2)	C14—C15—C20—C19	177.8 (2)
C3—C4—C5—O5	120.05 (18)	O7—C1—O1—C4	−177.88 (19)
O1—C4—C5—C6	−109.20 (19)	C2—C1—O1—C4	2.0 (2)
C3—C4—C5—C6	3.8 (2)	C3—C4—O1—C1	14.2 (2)
O5—C5—C6—O2	−98.2 (2)	C5—C4—O1—C1	126.26 (18)
C4—C5—C6—O2	20.9 (2)	C2—C3—O2—C6	151.80 (17)
O4—C7—C8—C13	179.6 (3)	C4—C3—O2—C6	41.7 (2)
O3—C7—C8—C13	−0.8 (3)	C5—C6—O2—C3	−39.8 (2)
O4—C7—C8—C9	−3.5 (4)	O4—C7—O3—C2	2.5 (3)
O3—C7—C8—C9	176.2 (2)	C8—C7—O3—C2	−177.11 (18)
C13—C8—C9—C10	0.4 (4)	C3—C2—O3—C7	69.5 (2)
C7—C8—C9—C10	−176.7 (2)	C1—C2—O3—C7	−175.54 (18)
C8—C9—C10—C11	0.8 (4)	O6—C14—O5—C5	−1.1 (3)
C9—C10—C11—C12	−1.1 (4)	C15—C14—O5—C5	179.92 (18)
C10—C11—C12—C13	0.1 (4)	C6—C5—O5—C14	−169.6 (2)
C11—C12—C13—C8	1.1 (4)	C4—C5—O5—C14	77.1 (2)
C9—C8—C13—C12	−1.3 (4)		

### Hydrogen-bond geometry (Å, º)

**Table:**

*D*—H···*A*	*D*—H	H···*A*	*D*···*A*	*D*—H···*A*
C2—H2···O6^i^	1.00	2.34	2.974 (2)	121
C3—H3···O4^ii^	1.00	2.51	3.356 (3)	142
C10—H10···O7^iii^	0.95	2.48	3.353 (3)	154

Symmetry codes: (i) *x*+1/2, −*y*+3/2, −*z*+1; (ii) *x*−1/2, −*y*+1/2, −*z*+1; (iii) *x*, *y*−1, *z*.
